# Short amylin receptor antagonist peptides improve memory deficits in Alzheimer’s disease mouse model

**DOI:** 10.1038/s41598-019-47255-9

**Published:** 2019-07-29

**Authors:** Rania Soudy, Ryoichi Kimura, Aarti Patel, Wen Fu, Kamaljit Kaur, David Westaway, Jing Yang, Jack Jhamandas

**Affiliations:** 1 Department of Medicine (Neurology), Neuroscience and Mental Health Institute, University of Alberta, Edmonn, AB Canada; 2 Department of Biochemistry, University of Alberta, Edmonton, AB Canada; 3 Center for Prions and Protein Folding Diseases, University of Alberta, Edmonton, AB Canada; 4 Center for Liberal Arts and Sciences, Sanyo-Onoda City University, Yamaguchi, Japan; 5 Chapman University School of Pharmacy, Irvine, CA USA; 6 Faculty of Pharmacy, Cairo University, Cairo, Egypt

**Keywords:** Drug development, Neurological disorders

## Abstract

Recent evidence supports involvement of amylin and the amylin receptor in the pathogenesis of Alzheimer’s disease (AD). We have previously shown that amylin receptor antagonist, AC253, improves spatial memory in AD mouse models. Herein, we generated and screened a peptide library and identified two short sequence amylin peptides (12–14 aa) that are proteolytically stable, brain penetrant when administered intraperitoneally, neuroprotective against Aβ toxicity and restore diminished levels of hippocampal long term potentiation in AD mice. Systemic administration of the peptides for five weeks in aged 5XFAD mice improved spatial memory, reduced amyloid plaque burden, and neuroinflammation. The common residue SQELHRLQTY within the peptides is an essential sequence for preservation of the beneficial effects of the fragments that we report here and constitutes a new pharmacological target. These findings suggest that the amylin receptor antagonism may represent a novel therapy for AD.

## Introduction

Alzheimer’s disease (AD) is the most common form of dementia that affects over 44 million individuals worldwide, and its prevalence of this condition continues to rise^1^. One of the defining features of AD is the presence of soluble oligomers of amyloid beta (Aβ) protein that aggregate into extracellular fibrillary deposits known as amyloid plaques. Progressive accumulation of intra- and extracellular Aβ within brain regions, that are critical for memory and cognitive functions, is linked to the neurodegeneration observed in this condition^2–4^. Progressive accumulation of Aβ is an early pathological event in AD and may precede clinical symptom onset by 15–25 years^5^. Recent clinical trials aimed at reducing the levels of Aβ, either through increasing its brain clearance using Aβ vaccine-based therapies, or inhibiting its generation by blocking the involved secretase enzymes, have been largely unsuccessful^6,7^. Thus as yet, of the four FDA-approved therapies for AD, none are disease-modifying.

One potentially promising approach for the treatment of AD includes targeting specific receptors that serve as mediators of the toxic effects of Aβ oligomers. Multiple receptors (the p75NTR receptor, scavenger receptors such as SCARA1/2, neuronal nicotinic acetylcholine receptors), have been implicated in mediating Aβ-induced disruption of neuronal and synaptic processes in AD and thus identified as potential drug targets for developing anti-Aβ therapies, although as yet none have fulfilled this goal^8,9^. Nonetheless, identification of a target that is implicated in the three key aspects of Alzheimer disease pathogenesis, i.e. neuronal loss, inflammation and vasculopathy, could offer a promising avenue for the development of therapeutics aimed at mitigating disease progression.

Emerging lines of evidence have highlighted the role of amylin receptor (AMY) as a putative target for the deleterious effects of Aβ in the context of AD^10^. Amylin receptor is a Class B G-protein-coupled receptor comprised of heterodimers of calcitonin receptor (CTR) and one of three receptor activity modifying proteins (RAMP1-3) that generate multiple subtypes of amylin receptors, AMY1-3^11,12^. Amylin receptor antagonist, AC253, is a 24-amino acid peptide, originally derived from 8-32 fragment of salmon calcitonin hormone^13^. Data from our group and others demonstrates that amylin receptors are abundantly expressed on neurons, microglia, and vasculature, three core elements implicated in the AD pathology^10,14–16^. Furthermore, Aβ-induced dysfunction and death of neurons that are preferentially affected in AD is attenuated by administration of amylin receptor antagonists, AC253 and AC187^17–19^. Interestingly, AC253 also effectively reverses the impairment of Aβ- or human amylin (hAmylin)-induced depression of hippocampal long-term potentiation (LTP), a recognized cellular surrogate of memory^20^. Most importantly, a recent study demonstrated that intracerebroventricular (icv) infusions of AC253 or intraperitoneal administration of the brain penetrant cyclized AC253 (cAC253), improved age-dependent deficits in spatial memory and learning in transgenic AD mice without gross adverse effects^21^. These antagonists improved synaptic markers along with suppression of microglial activation and neuroinflammation^21^. Other studies have reported that peripheral administration of amylin or its synthetic analog, pramlintide, resulted in improved spatial memory in mouse models of AD^22,23^. The improvement in behavioral measures and accompanying reduction in amyloid burden in the brain in these studies was attributed to an efflux of brain Aβ (including monomers and small oligomers) into the blood. Thus, the presence of amylin peptides in the circulation was postulated to serve as a “peripheral sink” for the egress of amyloid across the blood brain barrier and deemed to involve amylin receptors located on endothelial cells^23^. Collectively, these studies identify the amylin receptor as a viable and potentially promising target for the development of AD therapeutics.

In order to optimize AC253 based peptides for AD therapy, we generated shorter peptide fragments based on the AC253 sequence for additional translational studies. Shorter peptides offer several advantages over longer sequences: higher stability and selectivity, better toxicity profile, significant brain penetration when administered systemically and a lower cost for both small- and large-scale synthesis and purification^24–26^. Hence, we screened an AC253-based peptide fragment library and identified two promising shorter peptides, R5 (SQELHRLQTYPR), and R14 (LGRLSQELHRLQTY), which demonstrate high affinity binding to the amylin receptor subtype 3 (AMY3) and also recapitulate neuroprotective properties of the full length AC253. In experimental *in vitro* and *in vivo* transgenic AD models, these peptide fragments show a significant improvement in memory and learning, and an attenuation of some characteristic features of AD pathology.

## Results

### Identification of short peptide fragments that selectively bind to amylin receptor

In order to identify shorter peptides with selective recognition and binding to amylin receptor, enhanced metabolic stability and brain penetrability than the linear full length peptide, we designed a peptide library comprised of 14 different sequences, namely, R1-R14. Fragments (R1−R13) are 12 amino acids in length and peptide R14 is 14 amino acids. The initial fragment comprised the first 12 amino acids from the N-terminus of AC253 sequence, and subsequent fragments derived from shifting one amino acid at a time, as depicted in Supplemental Fig. S1A. The library was synthesized on non-cleavable cellulose membrane (aminoPEG500) using SPOT synthesis, where the C-terminus of the peptide was attached to the surface of the amino-PEG500 cellulose membrane through β-ala spacer as described previously^27^. Each amino acid was added to the free amino functional group using a stepwise Fmoc-SPPS procedure. Each peptide was synthesized in duplicate at approximately 50 nmol on a spot on the membrane with a diameter of 4 mm (Supplemental Fig. S1B). Since our previous studies identified AMY3 receptor subtype as the preferential target for the direct actions of Aβ (and hAmylin) at the level of the cell membrane^10^, we targeted this receptor isoform in the current study. A peptide library membrane was incubated with green fluorescent protein (GFP) labeled AMY3-expressing HEK293 cells (HEK-AMY3) to identify the highly binding sequences (Supplemental Fig. S1B). The relative binding affinities of peptide fragments were determined through measuring and plotting the net fluorescence intensity of the bound GFP labeled live cells on each spot as measured with a fluorescence Kodak imager (Supplemental Fig. S1C). Furthermore, to evaluate amylin receptor specificity of binding, the library was further screened against transfected CTR, and Wild-type HEK293 cells (HEK-WT). (Supplemental Fig. S1C).

The screening identified several peptide fragments that demonstrated significant specific binding to HEK-AMY3 cells compared to either HEK-WT or HEK-CTR cells (Supplemental Fig. S1C,D). Fragments from the N-terminus domain showed higher affinity binding to the AMY3 receptor compared to those generated from the C-terminal region. Among the array of peptide fragments, peptides R5, and R14 were selected as demonstrating the highest specific binding to HEK-AMY3 expressing cells (Supplemental Fig. S1D). Both peptide R5, and R14 bind with 2- fold more affinity to HEK-AMY3 cells based on intrinsic fluorescence measurement compared to HEK-WT cells, which confirmed their specificity for the amylin receptor and thus they were chosen for further investigation.

### R5 and R14 fragments showed significant binding to AMY3 receptor and are neuroprotective against Aβ-induced neuronal cell death *in vitro*

Peptide fragments R5, and R14 that demonstrated specific binding to AMY3 were synthesized for further *in vitro* studies, and R11, a peptide sequence with minimal binding to AMY3 expressing cells, was used as a negative control. Synthetic peptides were obtained in high yields (>75%), and purity exceeding 95% (Supplementary Table S1).

**Figure 1:**
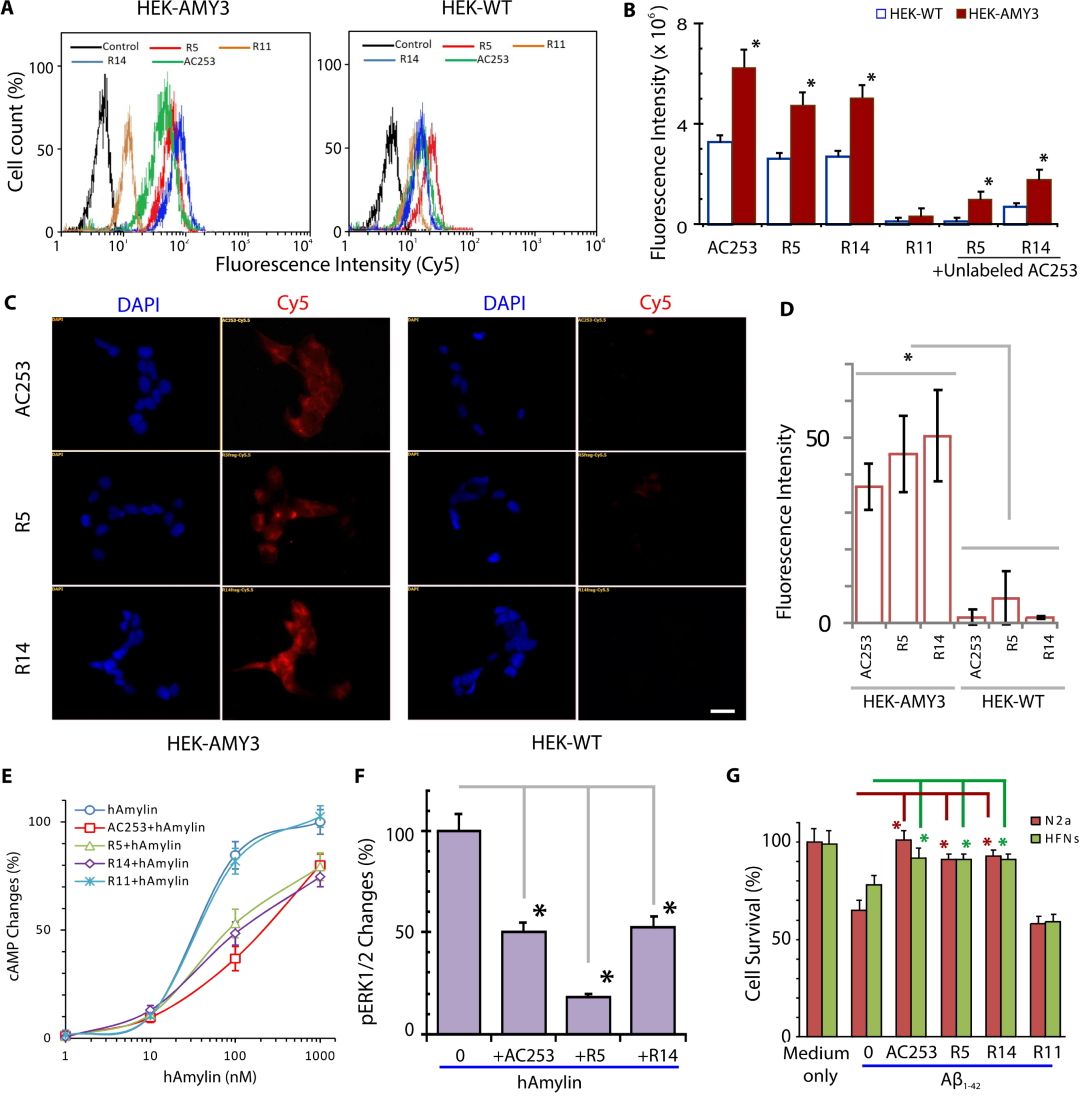
Fragments R5 and R14 retain amylin receptor antagonist and neuroprotective properties against Aβ toxicity. (**A**) Flow cytometry histograms showed that Cy5.5 labeled AC253, R5 and R14 have enhanced specific binding to AMY3 cells (HEK-AMY3) compared to wild type HEK cells (HEK-WT). R11 showed minimal binding activity. (**B**) Bar graphs showing quantification of flow cytometry uptake of Cy5.5 labeled AC253, R5 and R14 peptides in HEK-AMY3 compared to HEK-WT cells. There was no significant difference between AC253, R5 and R14. The uptake of R5 and R14 was significantly reduced in presence of unlabeled AC253 peptide (competitive binding inhibitor for amylin receptor). (Data is expressed as mean ± SE, n = 6, one-way analysis of variance followed by Tukey’s test, *denotes significant difference between HEK-WT and HEK-AMY3 cells, p < 0.05). (**C**) Representative fluorescence microscopy images showing Cy5.5 labeled peptides binding to HEK-AMY3 cells compared to HEK-WT cells (scale bar, 10 μm, DAPI = blue nuclear stain). (**D**) Bar graphs summarize the average fluorescent intensity in HEK-AMY3 and HEK-WT cells incubated with Cy5.5 labeled AC253, R5 and R14 peptides. The fluorescence intensity is significantly increased in HEK-AMY3 compared to HEK-WT cells. **(E)** R5 and R14 peptides (and AC253), but not R11, inhibited the increased levels of cyclic adenosine-monophosphate (cAMP) evoked by human amylin (hAmylin) activation of AMY3 receptors on HEK-AMY3 cells. Graphs shows changes in cAMP levels in HEK-AMY3 cells after exposure to different concentrations of hAmylin in presence of peptides (10 µM). (**F**) In HEK-AMY3 cells, AC253, R5 and R14 peptides (10 μM) reduce increases in phosphoERK1/2 evoked by hAmylin (1 μM) (n = 3,*p < 0.05). (**G**) Both fragments block the effect of oligomeric Aβ_1–42_ (10 µM)-induced cell death in primary cultures of human fetal neurons (HFNs) and N2a cells as shown with MTT cytotoxicity assay (n = 5, *p < 0.05).

Next, we labeled R5, R14 peptides with Cy5.5 to examine their *in vitro* binding efficacy and specificity compared with AC253 in HEK-AMY3 cells using flow cytometry and fluorescence microscopy (Fig. 1A,C). Flow cytometry data (Fig. 1A,B) revealed that R5, and R14 both displayed enhanced binding to HEK-AMY3 cells similar to AC253 with mean fluorescence intensity arbitrary units of 70 ± 10, 50 ± 15, and 20 ± 5, respectively. In HEK-WT cells, R5, R14 and AC253 demonstrated reduced binding and uptake compared to that for HEK-AMY3 cells, thus confirming AMY3 binding specificity. The R11 fragment showed minimal binding to AMY3, further supporting our library screening results. Uptake of R5 and R14 into HEK-AMY3 cells was competitively inhibited when cells were pre-incubated with unlabeled AC253 with mean fluorescence intensity of 1 × 10^6^, 1.5 × 10^6^ for R5, and R14, respectively, thus supporting amylin receptor based peptide cell uptake (Fig. 1B). With fluorescence microscopy, we observed strong binding of R5 and R14 fragments to the cell membrane of HEK-AMY3 cells compared to AC253, with minimal binding in HEK-WT cells (Fig. 1C,D).

We next examined the antagonistic properties of R5, and R14 at the AMY3 receptors and whether these peptides showed neuroprotective properties against Aβ toxicity. Human amylin is a potent agonist at amylin receptor stimulating cAMP production in cells^10^. In the first *in vitro* bioassay, we examined ability of R5 and R14 to block the hAmylin-evoked cAMP generation in HEK-AMY3 cells. R11 peptide served as a negative control. Results showed that R5 and R14, but not R11, peptides blocked the cAMP increases in a dose-dependent manner (Fig. 1E). Additionally, these peptides also blocked downstream activation of ERK1/2 signaling pathway (Figs 1F and S4A), which is activated by hAmylin and Aβ_1–42_ in HEK-AMY3 cells^10^. In a second *in vitro* assay, we examined whether peptides R5, and R14 could protect human fetal neurons (HFNs) and N2a (mouse neuroblastoma cell line) from Αβ_1–42_ induced cytotoxicity. Using the MTT assay, we observed that in both cell cultures, R5 and R14 peptides were equally effective as the full length AC253 in attenuating cell death induced by Aβ_1–42_. Cell survival was increased from 70% to 90% after pretreatment of cultures with fragments R5 and R14; in contrast, R11 did not show any effect in attenuating Aβ neuronal toxicity (Fig. 1G). Thus, these findings validated the library screening results, and indicated that the two fragments not only retained their antagonist activity at the amylin receptor, but also demonstrated neuroprotective properties against Aβ toxicity as seen with the full length AC253 peptide.

### Fragment R5 has significant blood brain barrier permeability *in vivo* after ip administration and its brain uptake correlates to the degree of amylin receptor expression

**Figure 2:**
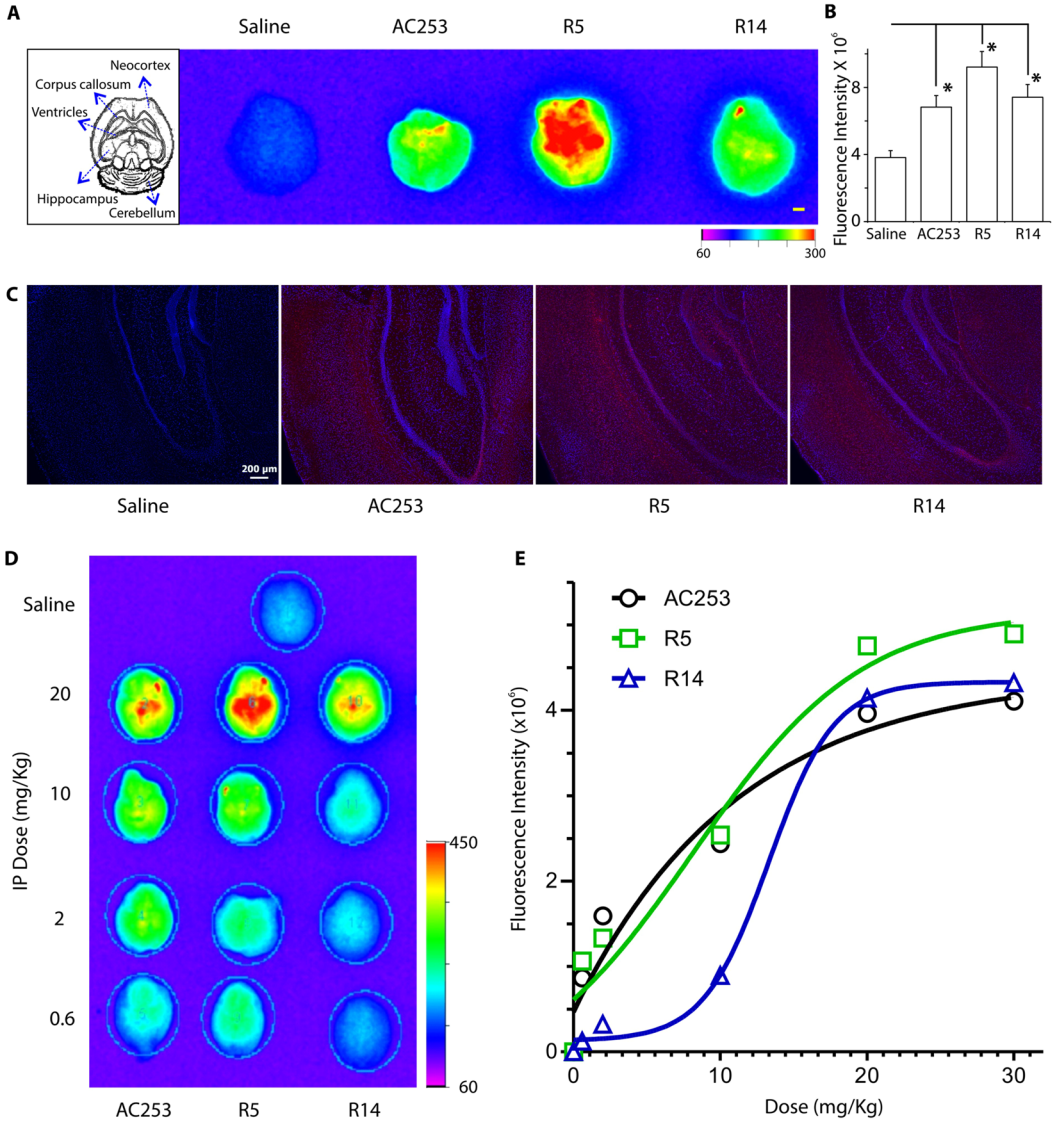
Fragments R5, R14 and AC253 demonstrate brain permeability *in vivo*. (**A**) Representative *ex vivo* fluorescence brain images for Cy5.5 labeled peptides (0.1 mmole in 200 μl normal saline) demonstrating their accumulation in the mouse brain 2 h after intraperitoneal (ip) injection. Scale bar = 1 mm. (**B**) Histograms showing brain fluorescence intensity was significantly increased 2 h following a single ip injection with labeled peptides (AC253, R5 and R14) compared to saline injection, but there was not difference between these peptides. (Mean ± SE, n = 10 in each group, one-way ANOVA followed by Tukey’s test, *p < 0.05). **(C)** Brain sections from *ex vivo* experiments in (**A**) showing AC253, R5 and R14 fluorescent labeling with Cy5.5 (red) within the hippocampal region, and nuclear staining with DAPI (blue). Scale bar = 200 µm. (**D**) *Ex vivo* fluorescence brain images showing dose-dependent accumulation of R5, R14 and AC253 after a single ip injection with different doses of the peptides in 200 µL saline. **(E)** Quantification of brain accumulation of labeled peptides at different concentration (n = 3 for each concentration).

Blood brain barrier (BBB) penetration can limit the potential of long sequence peptides as therapeutic agents in central nervous system (CNS). Previously, we have reported that a cyclized form of AC253 peptide and to a lesser degree its linear form can both penetrate the BBB at therapeutically relevant levels, and are localized to the hippocampus and cortex, regions relevant to memory and learning processes^21^. Therefore, we examined the ability of Cy5.5 labeled peptide fragments (R5, and R14) to penetrate BBB in wild-type mice using NIR fluorescence *ex vivo* brain imaging. Brain fluorescence resulting from either R5 or R14 was assessed against full length AC253 2 h after a single intraperitoneal (ip) injection of peptides. In an earlier study, using LC-MSMS, we demonstrated the intact Cy5.5 labeled cAC253 was present in the mouse brain when injected ip and that the Cy5.5 label was not hydrolyzed off the peptide^21^. All three peptides had some ability able to penetrate the BBB, but that the fluorescence signal for peptide R5 was significantly higher in the brains of these mice compared to either R14 or AC253 with mean fluorescence intensity of 9 × 10^6^, 7.2 × 10^6^, 7.0 × 10^6^, respectively (Fig. 2A,B). Fluorescence signals were distributed throughout the cortex, but particularly strong within the hippocampal regions, where a very high density of amylin receptor expression has been reported^15^. Histological analysis of *ex-vivo* imaged brains further confirmed that our peptides mainly accumulated in the hippocampal region (Fig. 2C).

Next, we examined the pharmacokinetic profile and the proteolytic stability of R5, and R14 compared to AC253 *in vitro and in vivo*. We analyzed peptides fluorescence levels in wild-type mice that received 0, 0.6, 2, 10, 20 mg/kg as ip single dose after 2 h. Results demonstrate that accumulation of R5, and R14 peptides in the brain appears to be dose-dependent (Fig. 2D,E). Bio-distribution evaluation of R5, R14 compared to AC253 in different organs (liver, kidney, spleen, heart, and brain) was investigated 2 h after injecting 20 mg/kg peptide. *Ex-vivo* fluorescence signals from tissues indicated that all peptides were distributed within all organs examined although uptake in the lung, spleen, and heart was considerably less than that in the kidney and the liver, which showed a strong NIR fluorescence intensity likely reflecting renal and hepatic clearance of the peptide (Supplemental Fig. S2A,B).

Subsequently, we investigated the proteolytic stability of R5, and R14 compared to AC253. R5 and R14 showed comparable serum stability with half-life of 1 h and 1.5 h, respectively, which is comparable to AC253 (1 h) (Supplemental Fig. S2C). By assessing the main degradation fragments for both peptides using MALDI-TOF, we found both peptides to be cleaved at the basic arginine amino acids.

In the present study, we also compared the brain uptake of R5 to that of davalintide, a second-generation synthetic amylinomimetic peptide possessing pharmacological properties superior to those of its congener, pramlintide^28^. We used heterozygous CTR mice that exhibit 50% CTR expression (“het CTR”) and hence 50% reduction in the functional amylin receptor^29^, and Wild-type mice, both groups receiving ip injections of either R5, or davalintide (0.1 mmole). Imaging of the intact brain at 2 h post-injection showed greater brain permeability of R5 compared to davalintide. As anticipated, CTR (amylin receptor) hemizygous mice showed significantly reduced peptide concentrations in comparison to the Wild-type mice (Supplemental Fig. S3).

### R5 and R14 fragments, but not R11, antagonize Aβ and hAmylin-induced depression of hippocampal long-term potentiation (LTP)

**Figure 3:**
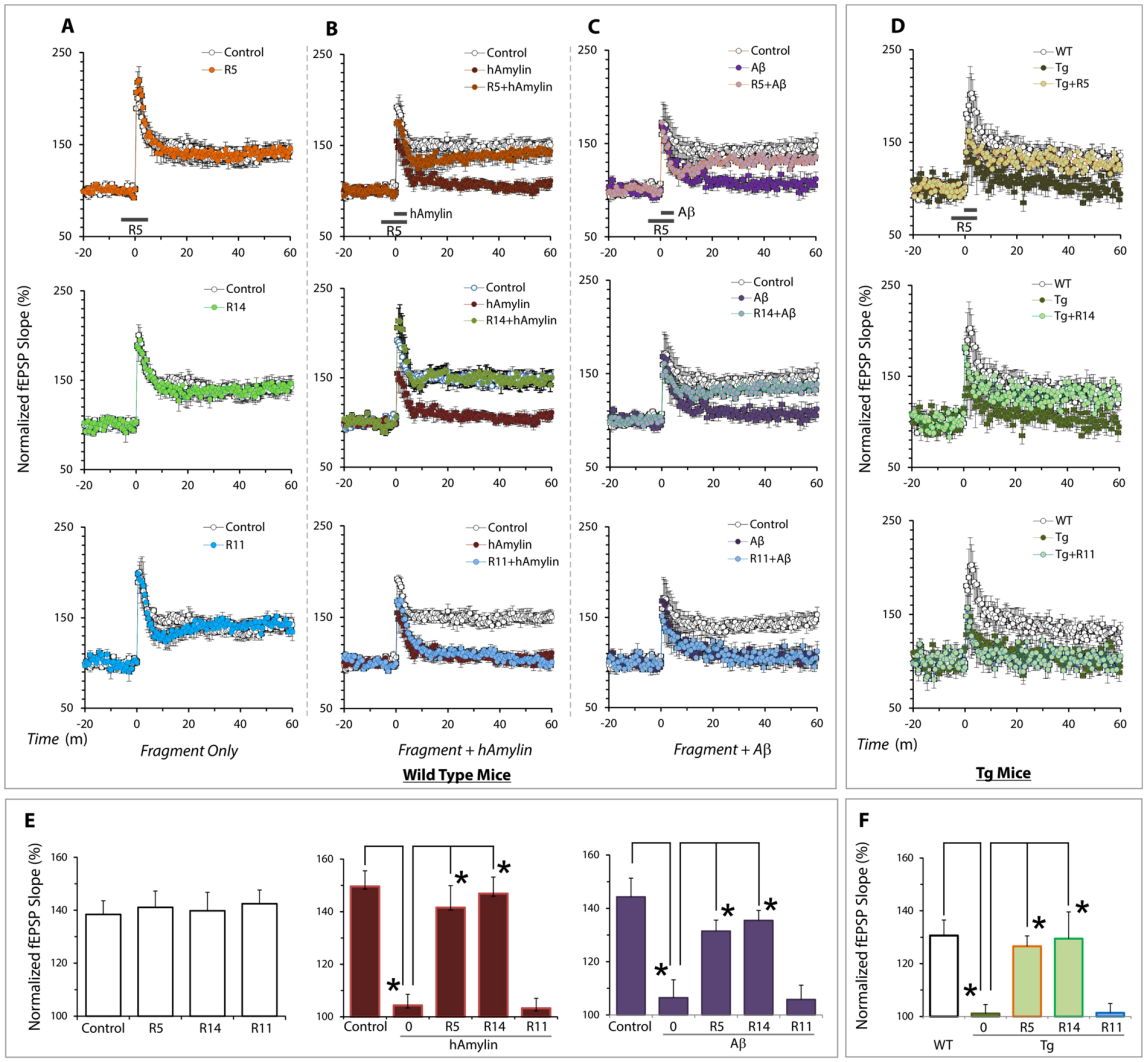
R5 and R14 peptides improve hippocampal long term potentiation (LTP). (**A)** The fragments R5, R14 and R11 (250 nM) alone did not impair LTP in hippocampal slices from wild type mice. (**B**) R5 and R14 but not R11 reverse human amylin (50 nM) and **(C)** Aβ_1–42_ (50 nM)-evoked reduction of LTP. (**D**) In hippocampal brain slices from 8 month old of TgCRND8 mice in which LTP is chronically depressed, R5 and R14, but not R11, restored LTP levels comparable to those observed in age-matched wild type littermate control mice. **(E)** Summary of the effects of R5, R14, and R11 fragments on hippocampal LTP in wild type mouse and (**F**) TgCRND8 AD mice. All data are presented as mean ± SEM. (n = 6 recordings for each group*p < 0.01, **p < 0.05; one-way ANOVA followed Tukey’s test).

We examined whether the peptide fragments were capable of influencing Aβ- or hAmylin-induced reduction of hippocampal LTP in mice. Exposure of hippocampal slices from wild-type mice to hAmylin (50 nM) or Aβ (50 nM) depressed LTP induced by a weak tetanization protocol at the CA1 region as previously reported^20,30^. To determine whether peptides affected hAmylin or Aβ- induced depression of LTP, we applied 250 nM R5 or R14 continuously for 5 min prior to exposure of the hippocampal slices to 50 nM hAmylin or Aβ and the subsequent LTP induction. Both R5 and R14, but not R11 peptide, reversed hAmylin and Aβ-induced depression of hippocampal LTP, while application of any of the three peptides alone did not affect basal hippocampal LTP levels (Fig. 3A–C). The composite data from these experiments are shown in Fig. 3E. In a prior study we had demonstrated that in aged TgCRND8 mice, which demonstrate an over-expression of Aβ, AC253 substantially improved the normally depressed basal levels of hippocampal LTP in these mice^21^. We therefore sought to determine whether peptide fragments were also capable of improving LTP levels in the same TgCRND8 AD mouse model. In 8 month old TgCRND8 mice, R5 and R14, but not R11, applications resulted in significant increase in LTP to levels approaching those recorded from age-matched wild type littermates (Fig. 3D,F). Importantly, doses of the short peptide fragments used in these experiments were equimolar to those used for AC253 and pramlintide in our previous studies^20,30^.

### Treatment with R5 peptide improves spatial memory and features of AD pathology in 5XFAD mouse model

**Figure 4:**
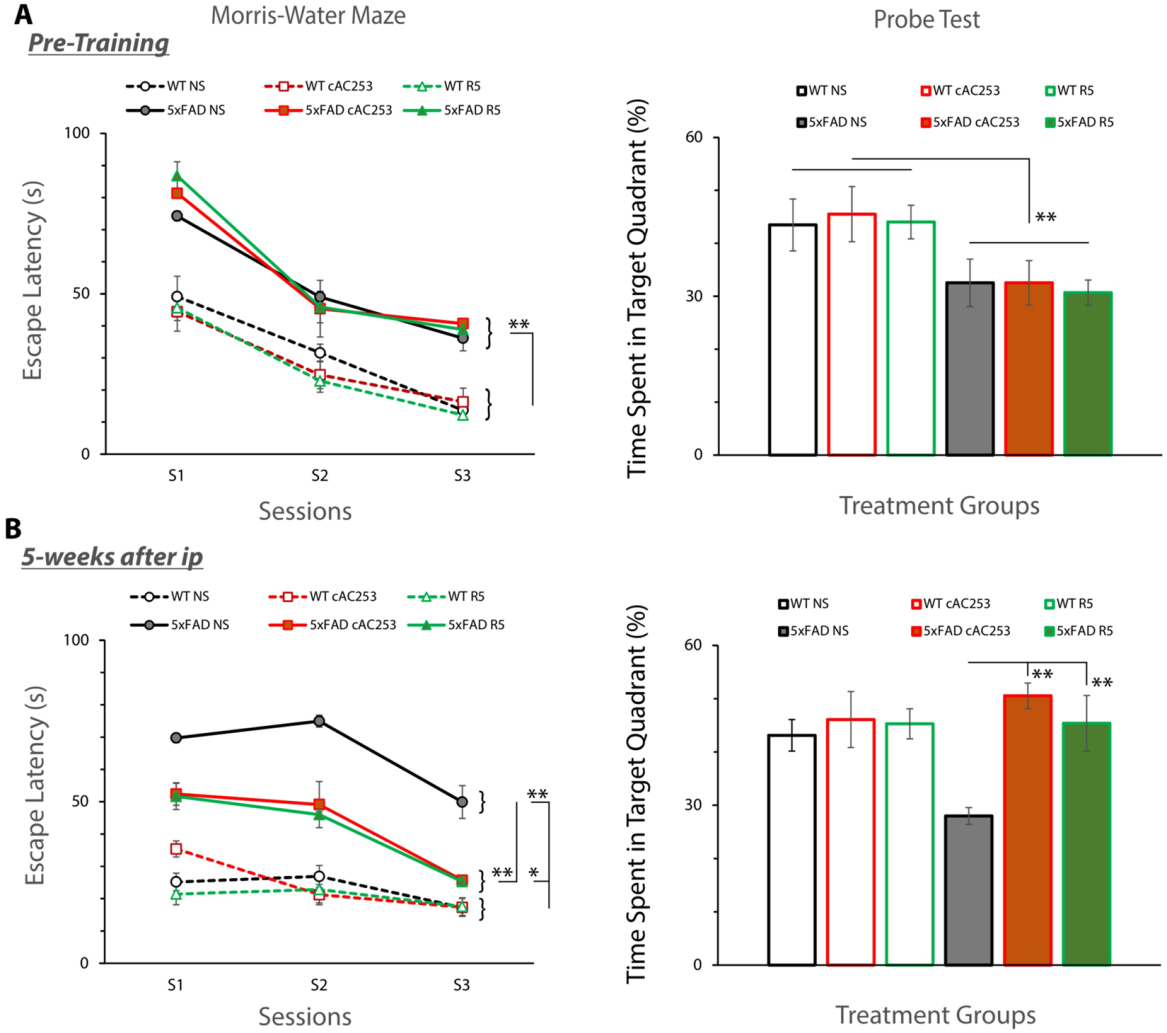
Systemic administration of R5 peptide improves cognitive function in transgenic AD mice. (**A**) Morris Water Maze (MWM) testing and Probe Test show significant cognitive function impairment in escape latencies and quadrant preference in 6 month old 5xFAD mice compared to their age-matched wild type (WT) littermate control mice before initiation of treatment. However, no difference was observed within the 5XFAD or WT groups destined to receive intraperitoneal (ip) injections of either R5, cyclized AC253 (cAC253) or normal saline (NS). (**B**) 5XFAD mice that either received R5 or cAC253 ip injections three times a week for 5 weeks demonstrated a marked improvement in escape latencies over those 5XFAD littermates receiving NS. In Probe trials, 5xFAD mice that were treated with R5, or cAC253 also showed preference for the target quadrant where the platform had been located. Wt littermate controls showed no memory deficits with either groups (n = 9 mice in each group; *p < 0.05, **p < 0.01).

To evaluate the effect of amylin treatment on learning and spatial memory, we employed established methods using the Morris Water Maze in an aggressive mouse model of AD, the 5XFAD. The 5XFAD mouse model is widely used as it recapitulates many AD related phenotypes with a relatively early onset and aggressive presentation of pathology and cognitive impairment^31^. The initial amyloid deposition begins by 2 months, and by 6 months the brain is characterized by the presence of a large number of amyloid plaques and other features of AD pathology^31^. At 6 months of age, these mice showed a significant difference in spatial memory, as measured by escape latencies, compared to wild-type mice (Fig. 4A). We therefore chose this mouse model for our *in vivo* studies to examine the efficacy of R5 peptide *after* the onset of cognitive deficits and AD pathology.

In addition to testing the R5 peptide, we also used cAC253 as a comparator peptide since we have previously shown it to have superior blood brain barrier penetration compared to its linear form. The determination of injection amount of R5 (200 µg/kg) was based on achieving equimolar concentrations as the full length peptide, cAC253^21^ and prior studies that used pramlintide injections^22,23^. The normal mouse circulated endogenous amylin level is 0.7 ± 0.4 pmol/L in plasma^32^ and based upon our *in vitro* data (Fig. 1E) unlikely to perturb the function of peptides *in vivo* at these concentrations. After 5 weeks of treatment with ip injections of R5 and cAC253 three times a week, 5XFAD mice showed a marked improvement in spatial memory compared to the transgenic littermates receiving sterile saline (Fig. 4B); Age-matched wild-type control mice showed no alterations in the memory task with systemic administration of either AC253, R5 or saline (Fig. 4B). Additionally, 5XFAD mice that were treated with cAC253 or with R5 showed improved retentive memory for location of the target quadrant (Probe Test) compared to transgenic mice receiving saline (Fig. 4A,B). None of the mice receiving cAC253 or R5 showed any signs of off-target effects (e.g., sedation, impairment of gait, abnormal feeding or drinking behavior, weight loss, changes in gross appearance such as hair loss, and lack of grooming) throughout the 5 weeks of treatment, and no significant changes in body weight.

**Figure 5:**
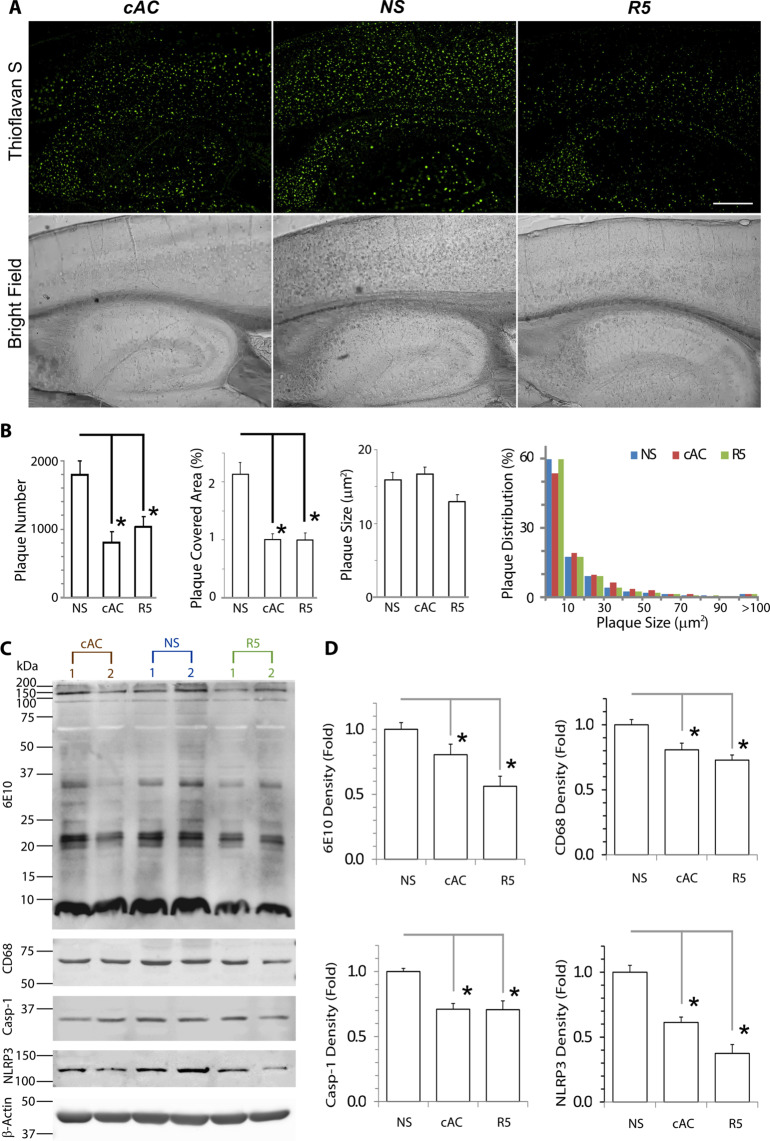
R5 peptide administration attenuates amyloid pathology and neuroinflammation in AD mice. (**A**) Brain amyloid plaques are significantly reduced after 5 weeks of ip injections of the R5 fragment or cAC253 in 5XFAD mice. Scale bar = 1000 μm. (**B**) Quantitative analysis revealed brain amyloid plaque number and density were significantly reduced in AD mice compared to normal saline (NS) control group (*p < 0.05). **(C)** Composite of Western blots showing amyloid proteins (Aβ, 6E10), CD68 (to identify activated microglia), and markers of inflammasome activation and neuroinflammation (Caspase-1, NLRP3) were all significantly reduced after a course of cAC253 or R5 ip injections compared to normal saline (NS). The full gels for CD68, Caspase-1 and NLRP are provided under Supplementary Information (Supplementary Fig. S5). Data shown in histograms (**D**) are normalized to β-actin signal (n = 6, *p < 0.05).

We initiated treatment of 5XFAD and wild-type control mice at 6 month age at a time point when they had also developed very significant amyloid burden in addition to the spatial memory deficits noted above (Fig. 4A). Compared with saline treated controls, a 5-week treatment regimen (ip injection three times a week) with either cAC253 or the shorter peptide (R5) significantly reduced amyloid pathology in the cortex, hippocampus and thalamus (Fig. 5A). There was significant reduction in the amyloid plaque numbers, and the overall amyloid burden (as judged by the area covered by plaques) in these brain regions in the peptide treated mice (Fig. 5B, p < 0.05). Western blot data also showed significant decrease in Aβ proteins from cortical tissue (Fig. 5C, p < 0.05). Furthermore, activated microglia CD68, the inflammasome NLPR3 and caspase-1, which are markers of neuroinflammation that is observed in AD pathology, were also significantly attenuated in 5XFAD that received either cAC253 or R5 peptides. (Fig. 5C,D, p < 0.05).

## Discussion

**Figure 6:**
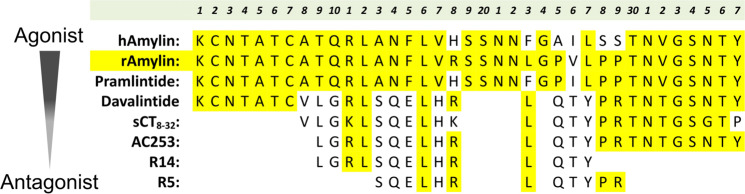
Alignment and comparison of sequences for amylin peptides. The amylin peptide sequences were aligned according to their known activity related to amylin receptor. The block of residues SQELHRLQTY comprises a common sequence amongst peptides that demonstrate antagonist activity at the amylin receptor and mediate their beneficial effects; this makes it an attractive potential therapeutic drug targets for AD.

Our results show that short sequence peptide fragments (Fig. 6), which are derived from the amylin antagonist, AC253, retain the antagonist activities of the parent peptide, are neuroprotective against Aβ toxicity in cell culture paradigms and improve hippocampal LTP in transgenic AD mice. Importantly, one of these fragments, R5, administered systemically not only improves spatial memory and learning in a transgenic AD mouse model, but also attenuates Aβ plaque load and neuroinflammation in the brain. This R5 peptide shows significant penetrability across the BBB and binding in the brain when administered systemically in mice. The improvements in spatial memory, reduction of amyloid load and neuroinflammation in the brain are noteworthy in two other respects. First, the improvement in spatial memory is apparent after a relatively short treatment in the AD mice (three ip injections per week for 5 weeks). Second, R5 treatment conferred benefit in 5XFAD mice that are 6-month old, an age at which both the AD pathology and behavioral deficits are well established. The latter observation is particularly relevant for clinical application since past and current anti-amyloid therapeutic interventions in AD patients after disease onset have been unsuccessful^6^. Thus, R5 or drugs based upon this peptide may represent an important advance in mitigating AD disease progression across a significant age span of the condition.

There are several potential mechanisms whereby R5 administration could improve measures of cognition and pathology in AD mice. First, R5 confers neuroprotection against Aβ in neuronal cell cultures and restores, in part, the disruption of hippocampal long term potentiation observed in AD mice as shown in the present study. Second, *in vitro* blockade of microglial amylin receptors, which we have shown to reduce recruitment of the inflammasome, NLRP3, and the secretion of pro-inflammatory cytokines^14^, explains our observations of attenuation of neuroinflammatory markers consequent to R5 administration in 5XFAD AD mice. Cytokines have been shown to upregulate APP processing and hence increase the generation of Aβ^33^ that could serve as a source for amyloid plaque formation. Thus blockade of microglial amylin receptors by R5 (and AC253) could attenuate Aβ production. Finally, administration of amylin based peptides have been shown to promote of efflux of Aβ from the brain through increased expression of LRP1^21,23,34^ and would explain the reduced amyloid burden following R5 treatment in our study.

Peptide-based therapeutics display favorable attributes because of their high potency and selectivity for their receptor targets, and often demonstrate a lower incidence of off-target effects compared with small molecule compounds^35^. As a step towards generating and identifying shorter peptide fragments based on AC253 sequence that demonstrate improved pharmacokinetic and/or pharmacodynamic profile, we used a cell-based peptide library screening assay^36^. This screening identified two peptides, R5, and R14, which retain not only the antagonist activity of the full length peptide, but also demonstrate comparable bioavailability, stability and brain penetrability. Our study also identifies regions within the AC253 peptide sequence that are likely responsible for amylin receptor binding and antagonistic activity (Fig. 6). Based upon the chosen cell-based screening assay, it appears that it is the short peptide sequences (R5 and R14) containing sequences derived from the N-terminal and middle rather than the C-terminal region of parent peptide (AC253) that demonstrate selective binding to the AMY3 receptor subtype (Supplemental Fig. S1). The three bioactive peptides (AC253, R5 and R14) share a common 10 amino acid sequence (SQELHRLQTY, Fig. 6), which not only confers the antagonistic activity at the AMY3 receptor, but also improves spatial memory and aspects of AD pathology in amyloid precursor protein (APP) over-expressing AD mice

The relationship of R5 and R14 peptides, which both demonstrate neuroprotective activity against Aβ evoked toxicity and improvement in spatial memory in transgenic AD mice, to other synthetic amylinomimetics such as pramlintide and davalintide deserves comment. Systemic injections of pramlintide have been shown to improve spatial memory in AD mouse models, a benefit that is presumably attributable to its amylin mimetic properties at the receptor^22,23^. However, in other experimental paradigms, pramlintide demonstrates an antagonist activity at the amylin receptor in a manner similar to AC253, R5 and R14 peptides^14,21^. Interestingly, davalintide, also an amylinomimetic, was synthesized to improve upon pramlintide’s pharmaceutical properties (short half-life and low bio-availability)^28^. However, our data indicate that davalintide, and in an earlier study pramlintide^21^, both demonstrate lower brain permeability after systemic administration than either cAC253 or R5. Thus, although longer half-life, stability and bioavailability are desirable attributes in the search for amylin based peptides as therapeutic agents for diabetes and other systemic conditions, in the case of AD, the ability of such peptides to cross the blood brain barrier is an important consideration for their therapeutic potential in the CNS. Hence, based in part on its superior brain permeability, we selected R5 to test its potential in reversing spatial memory deficits in transgenic AD mice. Salmon calcitonin (8–32), was the first peptide of the amylin family to be recognized for its antagonist activity at the amylin receptor and has been well recognized as having a slow receptor dissociation rate from receptors^37^. R5 sequence is embedded in a portion of the salmon calcitonin (8–32) sequence (Fig. 6) and would be consistent with demonstration of its antagonist activity at the amylin receptor that we have observed *in vitro* and *in vivo*.

In summary, we describe the synthesis and testing of short sequence amylin based peptides that demonstrate selective and full antagonist activity at the amylin receptor, are capable of attenuating the deleterious effects of Aβ on neurons and improve spatial memory and features of pathology in transgenic AD mice. The R5 peptide and its pharmacological characteristics described in this study offer many therapeutic advantages and could serve as platform for the design of novel non-peptide mimetics using computational modeling to treat AD.

## Methods

Detailed materials and methods are presented in the Supplementary Information. All *in vivo* experiments were carried out in accordance with the relevant laws and guidelines set by the Canadian Council for Animal Care and with the approval of the Animal Care Use Committee (Health Sciences) at the University of Alberta.

A shorter peptide library was derived from AC253 peptide sequence comprising 12–14 amino acid peptides. These shorter peptides were synthesized on a cellulose membrane using SPOT synthesis and screened for amylin receptor binding affinity with stable expressed amylin receptor GFP-positive cells. The effected shorted peptides from the library were labeled with a near-infrared fluorescent dye Cy5.5-NHS ester and further screened the receptor binding and uptake used AMY3 receptor and wild type control cells. To determine the activity of these shorter peptides to block AMY3 receptor signaling, cellular cAMP levels (measured using a parameter cyclic AMP assay kit, R&D Systems) and phosphorylation ERK1/2 (Western blot) were measured. We assessed neuroprotective properties of the peptide fragments against Aβ cytotoxicity *in vitro* using the MTT assay in N2a cell line and human fetal neurons (HFNs), which were prepared from 12- to 15-gestational week fetuses with approval of the Human Ethics Research Board at the University of Alberta as previously reported^17,19^.

*In vivo* brain penetration and pharmacokinetics of the peptides were determined in 6-month-old male or female wild-type (C57BL/6 background) and heterozygous CTR mice (het-CTR). Het-CTR mice (on C57BL/6J background) demonstrate a 50% depletion of CTR expression^29^. Excised mouse brains were imaged in Kodak imager at 2 h after ip injection with Cy5.5 labeled peptides at 0.2 nano-moles of peptides. These brains were then embedded in OCT and sliced into 20-μm slices, fixed with 4% paraformaldehyde, stained with DAPI and imaged Axio Zeiss fluorescent microscopy.

For electrophysiology experiment, hippocampal slices (400-μm thick) were prepared using a vibratome from wild type and TgCRND8 mice (8 month of age, male or female). For long-term potentiation (LTP) measurement, the stimulus strength was set to elicit 40–50% of the maximum fEPSP (field excitatory postsynaptic potential) amplitude and test pulses were delivered once every 30s. LTP was induced by 3-theta-burst stimulation protocol (each burst consisted of four pulses at 100 Hz with a 200-ms interburst interval). All drugs and chemicals were applied directly to the slice via bath perfusion.

For *in vivo* studies, 5XFAD mouse breeding stocks were obtained from the Jackson Laboratory (JAX #006554). Intraperitoneal injection (ip) administration of fragment R5 and cAC253 was carried out in 5XFAD mice and wild-type littermate control mice (both male and female). These mice were equally and randomly distributed into 6 groups (n = 10 for each group), and received either normal saline (NS), cAC253 or R5 fragment (200 µg/kg) i.p. injections 3 times a week starting at 6 months of age for 5 weeks.

The Morris Water Maze (MWM) and probe test were carried out for behavioral testing. The mice were trained for 3 days (4 trials per day) to find a submerged platform (target quadrant, TQ). The trial ended when a mouse found and climbed onto the platform within 120s. Mice were tested with inter-trial interval of 50 min. Memory was evaluated in probe trial, administered on day 4 as the first trial of the day. During probe trial, the platform was removed from the pool. Memory for the platform location was expressed as the percent of time spent in TQ.

After completion of treatments, the mouse brains were harvested and the right hemisphere was frozen for biochemical analysis (Western blot, ELISA), and the left hemisphere was fixed with PAF for further processed. The thicker brain sections were stained thioflavin-S for detecting Aβ plaques.

For statistical analysis, all values are present as means ± SD. Significance was determined using either student t test, or one-way analysis of variance, followed by Tukey’s test when appropriate, with Prism software (GraphPad Prism 5, GraphPad Software, San Diego, CA). p < 0.05 was considered significant.

## Supplementary information

Supplementary Information Text and Figures
