# Author Correction: Short amylin receptor antagonist peptides improve memory deficits in Alzheimer’s disease mouse model

**DOI:** 10.1038/s41598-022-10922-5

**Published:** 2022-04-28

**Authors:** Rania Soudy, Ryoichi Kimura, Aarti Patel, Wen Fu, Kamaljit Kaur, David Westaway, Jing Yang, Jack Jhamandas

**Affiliations:** 1 Department of Medicine (Neurology), Neuroscience and Mental Health Institute, University of Alberta, Edmonton, AB Canada; 2 Department of Biochemistry, University of Alberta, Edmonton, AB Canada; 3 Center for Prions and Protein Folding Diseases, University of Alberta, Edmonton, AB Canada; 4 Center for Liberal Arts and Sciences, SanyoOnoda City University, Yamaguchi, Japan; 5 Chapman University School of Pharmacy, Irvine, CA USA; 6 Faculty of Pharmacy, Cairo University, Cairo, Egypt

Correction to: *Scientific Reports* https://doi.org/10.1038/s41598-019-47255-9, published online 29 July 2019

The original Article contained an error in Figure 1A where the control trace for both the HEK-AMY3 and HEK-WT cells was duplicated. The original Figure 1 appears below.

**Figure 1:**
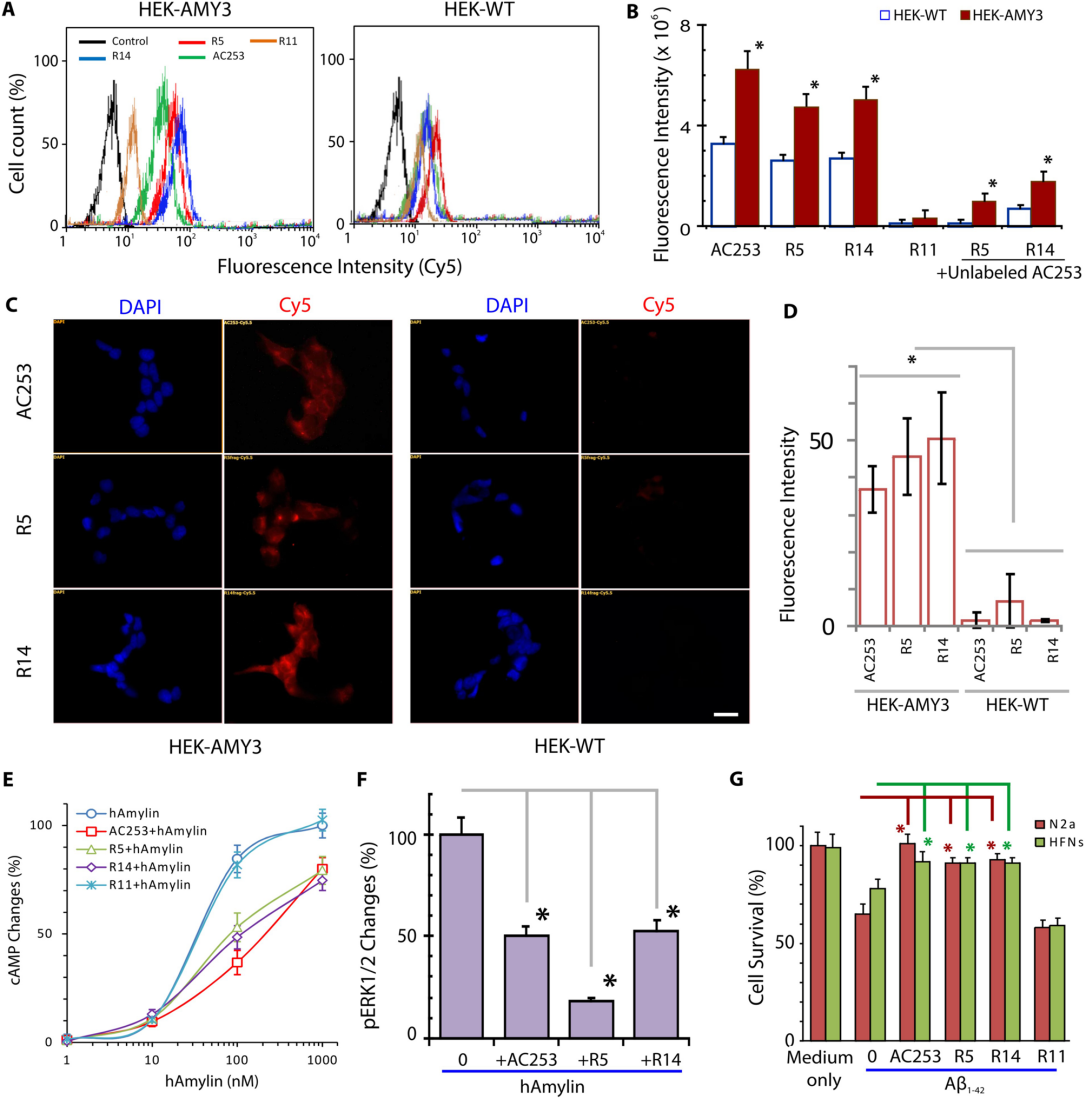
Fragments R5 and R14 retain amylin receptor antagonist and neuroprotective properties against Aβ toxicity. (**A**) Flow cytometry histograms showed that Cy5.5 labeled AC253, R5 and R14 have enhanced specific binding to AMY3 cells (HEK-AMY3) compared to wild type HEK cells (HEK-WT). R11 showed minimal binding activity. (**B**) Bar graphs showing quantification of flow cytometry uptake of Cy5.5 labeled AC253, R5 and R14 peptides in HEK-AMY3 compared to HEK-WT cells. There was no significant difference between AC253, R5 and R14. The uptake of R5 and R14 was significantly reduced in presence of unlabeled AC253 peptide (competitive binding inhibitor for amylin receptor). (Data is expressed as mean ± SE, n = 6, one-way analysis of variance followed by Tukey’s test, *denotes significant difference between HEK-WT and HEK-AMY3 cells, p < 0.05). (**C**) Representative fluorescence microscopy images showing Cy5.5 labeled peptides binding to HEK-AMY3 cells compared to HEK-WT cells (scale bar, 10 μm, DAPI = blue nuclear stain). (**D**) Bar graphs summarize the average fluorescent intensity in HEK-AMY3 and HEK-WT cells incubated with Cy5.5 labeled AC253, R5 and R14 peptides. The fluorescence intensity is significantly increased in HEK-AMY3 compared to HEK-WT cells. (**E**) R5 and R14 peptides (and AC253), but not R11, inhibited the increased levels of cyclic adenosine-monophosphate (cAMP) evoked by human amylin (hAmylin) activation of AMY3 receptors on HEK-AMY3 cells. Graphs shows changes in cAMP levels in HEK-AMY3 cells after exposure to different concentrations of hAmylin in presence of peptides (10 µM). (**F**) In HEK-AMY3 cells, AC253, R5 and R14 peptides (10 μM) reduce increases in phosphoERK1/2 evoked by hAmylin (1 μM) (n = 3,*p < 0.05). (**G**) Both fragments block the effect of oligomeric Aβ1–42 (10 µM)-induced cell death in primary cultures of human fetal neurons (HFNs) and N2a cells as shown with MTT cytotoxicity assay (n = 5, *p < 0.05).

The original Article has been corrected.

