# Supplementary material for: Short amylin receptor antagonist peptides improve memory deficits in Alzheimer’s disease mouse model
Source: Sci Rep. 2019 Jul 29;9:10942. doi: 10.1038/s41598-019-47255-9 (PMC6662706; doi:10.1038/s41598-019-47255-9)
Supplement: Supplementary file 1 — Supplementary Information Text and Figures [file 41598_2019_47255_MOESM1_ESM.docx]

# Short amylin receptor antagonist peptides improve memory deficits in Alzheimer’s disease mouse model.

Rania Soudy^1,^ ^†^ PhD, Ryoichi Kimura^4,^ ^†^ PhD, Aarti Patel^1, †^ PhD, Wen Fu^1, †^ MD, Kamaljit Kaur^5^ PhD, David Westaway^1,2,3^ PhD, Jing Yang^1,3^ MD, Jack Jhamandas^1,*^ MD, PhD

**Additional information**

**1. Detail Materials & Methods**

**2. Supplementary Table S1:** MALDI-TOF mass spectrum of peptide of the three selected peptides used in the study R5, R14, and R11.

**3. Supplementary Figures S1-4.**

Figure S1: Design and synthesis of AC253 based peptide library.

Figure S2: The comparison of bio-distribution for the fragments and their parent peptide.

Figure S3: The comparison of brain permeability for R5 and Davalintide.

Figure S4: The list of full membranes for Western Blot. .

**Additional information**

**1. Detail Materials & Methods**

**Materials**

All reagents and solvents were of analytical grade and used as received with no further purification. Rink amide resin (0.4mmol/g), (2-(6-chloro-1H-benzotriazole-1-yl)-1,1,3,3-tetramethylaminium hexafluoro phosphate), and Fmoc-amino acids were purchased from NovaBiochem (San Diego, CA). Fmoc-amino acids were supplied with the following side-chain protection: Fmoc–Asn(Trt)–OH, Fmoc–Arg(Pbf)–OH, Fmoc–Gln(Trt)–OH, Fmoc–His(Trt)–OH, Fmoc–Ser(O*t*Bu)–OH, Fmoc–Thr(O*t*Bu)–OH, Fmoc–Tyr(O*t*Bu)–OH. Trifluoroacetic acid (TFA), *N*,*N*-Diisopropylethylamine (*i*PrNEt), piperidine, and triisopropylsilane (*i*Pr3SiH) were purchased from Sigma-Aldrich (Canada). Cyanine5.5 NHS ester dye was purchased from Lumiprobe. Amino-PEG500 cellulose membrane derivatized with a polyethylene (PEG) purchased from (Intavis AG, Germany). *N*,*N*′-Diisopropylcarbodiimide (DIC), 1-Hydroxybenzotriazole hydrate (HOBT), *N*,*N*dimethylformamide (DMF), Dichlormethane (DCM), isopropyl alcohol (IPA) N-hydroxysuccinimide (NHS), *N*-methyl morpholine (NMM), trifluoroacetic acid (TFA), acetic acid, dimethyl sulfoxide (DMSO), triisopropylsilane, piperidine and all other reagents were purchased from Sigma-Aldrich (USA). Soluble oligomeric Aβ_1–42_ was purchased from rPeptide (Bogart, GA). Human amylin was purchased from American Peptide (Sunnyvale, CA). All peptides were stored as aliquots in microcentrifuge tubes at −80°C, and freeze-thaw cycles were limited.

**Peptide library screening**

A peptide array library derived from AC253 peptide sequence comprising 14 short peptide sequences namely, R1-R14 (R1-R13 are 12 amino acids, peptide R14 is 14 amino acids) was synthesized in duplicate on a cellulose membrane using SPOT synthesis as described previously ^33^. Briefly, peptide fragments were designed by selecting the first 12 amino acids from N-terminus, and shifting one amino acid at a time towards the C-terminus to yield 13 sequences R1-R13; peptide R14 comprised of the first 14 amino acids of the N-terminus region of AC253. The peptide array was synthesized on a PEG-500-derivatized cellulose membrane with a free amino terminal group using a semiautomatic robot AutoSpot ASP222 (Intavis AG, Germany). Synthesis of peptides was started by attaching first β-alanine residue (linker) to the cellulose membrane and subsequently peptides synthesized from the C-terminus. Fmoc protected amino acids (0.25 mM/mL) activated with HOBt and DIC were spotted on the membrane in 60 nL aliquots per spot by a robotic syringe, yielding a peptide loading of 0.4 μmol/cm^2^. After coupling of the Fmoc amino acid, the membrane was removed from the platform of robotic system and treated with acetic anhydride (2%) to cap any free remaining amino groups. Deprotection of Fmoc of coupled amino acid was performed using 20% piperidine in DMF. After deprotection, membrane was washed with DMF and IPA, air-dried and carefully repositioned on the robotic system to repeat the coupling cycles in order to complete the peptide sequence. At the end, all peptides were N-terminally acetylated. The final removal of side chain protecting groups was performed by treating the membrane with a cocktail of reagents, comprised of TFA (15mL), DCM (15 mL), triisopropylsilane (0.9 mL), and water (0.6 mL), for about 3 h. After extensive washing with DCM, DMF, and ethanol, the membrane was dried with cold air and stored in a sealed bag at −20 °C until use.

**Peptide Array-Cell Binding Assay**

To screen peptide library for amylin receptor binding affinity, transfected GFP-positive Human Embryonic Kidney 293 cells that express AMY3 receptor (CTR+RAMP3) as previously reported was used ^8^. For controls, wild type GFP-positive HEK293 cells and HEK293 cells that overexpress calcitonin receptor (CTR) cells were used. Cells were cultured in DMEM ([Invitrogen](http://www.jbc.org/cgi/redirect-inline?ad=Invitrogen)) with 10% FBS ([Invitrogen](http://www.jbc.org/cgi/redirect-inline?ad=Invitrogen)) and grown at 37 °C, 5% CO_2._ The peptide array membrane was incubated with 20 ml GFP-cells (75,000/ml) in serum free media for 3 h, and followed by washing unbound cells with PBS three times. The membrane was scanned at 465 nm excitation and 535 nm emission and the net fluorescence intensity of each peptide spot was quantified using Kodak IS2000MM Image station (GE Healthcare Systems/ART Inc). Duplicate peptide spots on each membrane and two scans of the same membrane were used to obtain the fluorescence intensity for each cell line the experiments were repeated twice to obtain the net florescence intensity.

**Peptide Synthesis and Fluorescence Labeling**

To validate peptide library results and investigate peptides amylin receptor binding and antagonistic activity, selected peptides were synthesized, fragments R5, and R14 were the most promising AMY3 binding sequences, while fragments R11 was selected as negative control. Peptide fragments, Davlintide and AC253 were synthesized on rink Amide MBHA resin at 0.1 mmol scale, using Fmoc/*t*Bu strategy by an automated synthesizer (Tribute, Protein Technology Inc., USA) as previously described. Fmoc chemistry, employing four equivalent of Fmoc protected amino acids and coupling agents like NMM and HCTU. The Fmoc group was removed using 20% piperidine in DMF (3 min X 2). Resin cleavage and removal of the amino acid side-chain protecting groups was undertaken by incubating the resin in cleavage cocktail of TFA/*i*Pr_3_SiH/H_2_O (v/v/v; 95/2.5/2.5) for 2 h at room temperature. The crude peptides were precipitated and triturated with cold diethyl ether, isolated (centrifugation), dissolved in 20% ACN (aq) containing 0.1% TFA and lyophilized. All peptides were purified on RP-HPLC using semi–preparative C18 (Vydac) column using gradient of 15-55% acetonitrile for 55 min with flow rate of 2ml/min. Water used in HPLC contained 0.05%TFA. Approximately 95-97% purity was gained for purified peptides, which was assessed using Vydac analytical C18 HPLC column. The peak containing the peptide was collected and lyophilized, retention times observed were 18.5 min, 19.2 min, and 18.3 min for R5, R14, and R11 peptides respectively. (Molecular mass of each peptide was evaluated by MALDI-TOF mass spectrometry. Calculated for R5, [M+H]^+^ 1527.5; found [M+H]^+^ 1526.5; calculated for R14, [M+H]+ 1713.1.6; found [M+H]+ 1712.1; calculated for R11, [M+H]+ 1351.6; found [M+H]+1351.8 (**Table S1**).

For peptides fluorescent labeling an extra β-alanine was added to the N-terminus of the peptide as a spacer. Subsequent labelling of AC253 and peptides with the near-infrared fluorescent dye Cy5.5-NHS ester (molecular weight 750.42D, λex 673 nm, and λem 707 nm) through the N-terminal amino group acylation was carried out as follows: Peptides (2 mMoleop) were dissolved in 0.5 mL of DMSO and 20 μL of Cy5.5 NHS ester dye was added (1.4 mg, 2 mM) with 30µl triethylamine and rotate on the shaker in the dark for 4 h at room temperature, then at 4 °C overnight. The labeled peptides were purified from unincorporated dye using RP-HPLC using gradient of 30-70% acetonitrile for 45 min with flow rate of 1.5 ml/min then 70-100 in 10 min, then back to 30% in 5 min. Retention times were 35 min, 34.5min, and 33 min for R5, R14, and R11 fragments respectively. HPLC purified peptides were pooled and lyophilized to give the fluorescent labeled peptides as blue powder in > 97% purity as assessed using Vydac analytical C18 RP-HPLC column. Stock solution of peptides (1 mM) were prepared in 100% DMSO, 100 µl aliquots were stored at -80 ºC, and before using DMSO was removed by lyophilization and the peptides were dissolved in the required buffers.

**Cell Culture and Relevant Assays**

For cAMP measurements, HEK-AMY3 cells were plated on 24-well plates overnight. Cells were then incubated with peptide fragments R5, R14 at conc 1 µM for 30 min. Then cells were stimulated for 30 min with hAmylin, over a concentration range (1 pM–10 µM). Cellular cAMP levels were measured using a parameter cyclic AMP assay kit (R&D Systems) according to the manufacturer’s instructions. Data was plotted, and non-linear regression was fitted with four parameters using Prism software (GraphPad Software, La Jolla, CA).

To investigate the antagonistic activity of peptide fragments against Aβ cytotoxicity in vitro, HFNs (human fetal neurons), human neuroblastoma SK-N-SH cells and N2a mouse neuroblastoma cells were used. Cells were seeded to 5000 cells/well in a 96-well plate in MEM-10% FBS and DMEM/OptiMEM-5% FBS for overnight. Cells in culture media were preincubated for 8 h with or without AC253 or fragments R5, R14, R11, and followed by treatment with Aβ_1–42_ for 24 h. At the end of treatment, 20 µl of 5 mg/ml methylthiazolyldiphenyl-tetrazolium bromide (MTT) was added to each well, and incubated at 37 °C for 3 h. Medium was removed, 100 µl of MTT solvent (isopropanol with 4 mM HCl) added to each well, and the plates were incubated for 30 min at room temperature on a rotating shaker. Plates were analyzed on a microplate reader at a 562-nm wavelength.

**Cell binding of peptides and uptake**

For flow cytometry studies, HEK-AMY3 cells were used to determine cell binding and uptake and binding of Cy5.5 labeled AC253, and R5, R14, R11 fragments. Cell were plated at a density of 1.5 × 10^5^ cells/well and cultured overnight in 12-well plates at 37 ºC. Peptides (5 µM) were added and incubated for 1 h at 37 ºC in serum free media. The culture media was then discarded, and cell monolayers were washed with PBS containing 2 mM EDTA and 0.5% BSA adjusted to pH 7.4 at 37°C, then cells were washed again with cold buffer and incubated with 0.25% trypsin to remove cell surface–associated fluorophores. Cells were then dispersed in FACS solution (10% FBS in PBS), and analyzed using a FACSCanto II flow cytometer (BD Biosciences, USA) selecting a detection window between 720 and 840 nm, and fluorescence histograms and dot plots were generated using FLOWJO software (Tree Star, Inc, USA). At least 10,000 gated events per sample were analyzed to develop the histogram. HEK-WT cells was used as negative control.

In a parallel experiment, that peptide cell binding was assessed using fluorescence microscopy. HEK-AMY3 or HEK-WT cells were grown on coverslips in 12-well plates at a density of 1.5 × 10^5^ cells/well until 50% confluence was achieved and subsequently incubated with Cy5.5 peptides R5, R14, AC253 (5 µM) diluted in culture medium at 37 °C for 1 hour. Then cells were washed three times with PBS, fixed in 4% paraformaldehyde in PBS for 10 min, and then mounted in DAPI mounting media. The cells were imaged using a Zeiss Axioplan-2 microscope (Carl Zeiss Microscope Systems, Toronto, ON, Canada) and AxioVision software (version 4.8) with identical photo settings.

For peptide cell uptake studies, 400,000 cells (HEK-AMY3, HEK-WT) were seeded into 6-well plates and cultivated for 24 h. The medium was replaced by 1 mL of fresh medium (without fetal calf serum). Cy5.5 labeled peptide was added to the cell culture (5 µM) and incubated for 30 mins. When the competitor was used, unlabeled peptide AC253 (10^−4^ M) was preincubated for 30 min. After incubation, the binding medium was aspirated and the cells were rinsed with ice-cold 0.01 M PBS/0.2% bovine serum albumin (pH 7.4) and centrifuged. This process was repeated twice to remove excess unbound peptides. The cell-binding capacity was reported as mean fluorescence intensity. After incubation, the cells were washed 3 times with 1 mL of PBS and subsequently lysed with 0.5 mL of 0.3 M NaOH. Fluorescence was quantified using a Kodak Image station, excitation filter 625/20 band pass, emission filter 700W band pass.

**Intracellular Signaling**

Intracellular signaling profiles were determined using in-cell Western blot techniques as previously described ^8^. HEK-AMY3 cells were seeded at 10,000 cells/well in a 96-well plate (Nalge Nunc Intl., Rochester, NY) in DMEM, 10% FBS, and Zeocin medium. After culturing for 12–16 h, cells were treated either or not with AC253 (10 μM) and hAmylin or Aβ_1–42_ in culture medium for time periods between 10 min and 30 h. Subsequently, cells were fixed with 4% paraformaldehyde for 20 min, permeabilized with 0.2% Triton X-100 PBS solution, blocked with Odyssey blocking buffer (LI-COR, Lincoln, NE), and stained with the following target antibodies. The phospho-p44/42 MAPK (ERK1/2, Thr-202/Tyr-204) rabbit polyclonal antibodies (Abcam, Inc. Cambridge, MA) and IRDye 800CW goat anti-rabbit antibody as secondary antibody, whereas Sapphire700 and DRAQ5 were used for cell number normalization (LI-COR). Plates were imaged using an Odyssey Infrared Imaging System (LI-COR), and the integrated intensity was normalized to the total cell number on the same well.

**Animal Models**

All experiments were carried out in accordance with the relevant laws and guidelines set by the Canadian Council for Animal Care and with the approval of the Animal Care Use Committee (Health Sciences) at the University of Alberta. Mice were housed individually under standard laboratory conditions (1212 h light/dark cycle, lights on at 0600 h) with a room temperature of 21°C. Water and food were available *ad libitum* unless otherwise indicated.

For experiments establishing the pharmacokinetics and brain penetration of the peptides, we used wild-type (C57BL/6 background) male or female 6 month old mice. Additionally, heterozygous CTR (het CTR) age-matched mice (C57BL/6J background) with a 50% depletion of CTR expression (*28*) were obtained using breeding pairs provided from Drs. RA Davey and JD Zajac (University of Melbourne, Australia). For *in vitro* LTP experiments, we used TgCRND8 mice ^35^ that were provided by Dr. David Westaway (University of Alberta). For *in vivo* studies, 5XFAD mouse breeding stocks were obtained from the Jackson Laboratory (JAX #006554).

**Drug administration for in vivo experiments**

For *in vivo* experiments, intraperitoneal injection (ip) administration of fragment R5 and cAC253 was carried out in 6 month old transgenic 5XFAD mice and wild-type littermate control mice (both male and female) obtained from Dr. David Westaway (University of Alberta). These mice were equally and randomly distributed into 6 groups and 10 mice assigned for each group, Tg-NS, Tg-cAC253, Tg-R5, Wt-NS, Wt-cAC253, Wt-R5. Randomized double blind control experiment was carried out for the whole *in vivo* experiment, i.e. the individual administering ip injections was blinded to the identity of the drug, and the individual testing behavior was blind to the treatment being administered. Mice received either normal saline (NS), cAC253 or R5 fragment (200 µg/kg) i.p. injections 3 times a week starting at 6 months of age for 5 weeks. Mice were housed under standard laboratory conditions (12/12-h light/dark cycle, lights on at 0600 h) with a room temperature of 21°C. Water and food were available ad libitum.

***Ex vivo* NIRF imaging**

For *ex vivo* imaging experiments and peptide brain uptake, we used age matched 6-month-old wild-type littermate (C57BL/6 background) mice. Mice were injected ip with Cy5.5 labeled peptides (R5, R14, AC253) at 0.2 nmoles of peptides in single dose in 200 µl normal saline. After 2 h the mice were then sacrificed to collect their brains, and image them in Kodak imager. Images were acquired with a Kodak Image station, excitation filter 625/20 band pass, emission filter 700 band pass. Images were captured with a CCD camera set to F stop = 0, FOV = 150, FP = 0. Exposure time was 3 min per image for NIRF image. Region of interest was drawn around the brain region and analyzed using the Kodak ID 3.6 software and the mean fluorescence intensity was recorded. For ex Vivo histological Study, excised brains were embedded in OCT and sliced into 20-μm slices, co-stained with DAPI in mounting medium. Florescence images were observed with Axio Zeiss fluorescent microscopy.

For peptide R5 brain uptake in comparison to Davlintide, wild-type littermate (C57BL/6 background) mice and heterozygous CTR knockdown mice (50% CTR expression level compared to wild type control mice and presumably with 50% AMY receptor expression level) was used (n=5 in each group). Mouse brains were extracted after 2h from a single injection and fluorescence in brains was quantified as mentioned above.

***In Vivo* Pharmacokinetics and Bio-Distribution Studies**

Mice were treated in a single dose with peptides, R5, R14 and AC253, at 0, 0.6, 2, 10 and 20 mg/kg in 200 µl normal saline, 3 mice for each dose. The mice were sacrificed after 2h and their brains were imaged usingKodak imager. For time based studies, mice were injected with 400 µg peptides and brains were collected at 0, 0.5, 2, 6, 24, and 48h, 3 mice per time point. For bio-distribution experiments, the different organs (heart, liver, lung, spleen, intestine, stomach, kidney and brain) were excised after perfusion with PBS after 2h single injection. The mean fluorescence intensity for each organ was imaged using Kodak imager and fluorescence was quantified, and analyzed as previously mentioned.

**Slice Preparation and Electrophysiology**

Brains were quickly removed from mice following decapitation, placed in a cold artificial cerebral spinal fluid (aCSF) on a vibratome chamber, and transverse sections cut through the hippocampus. The aCSF contained (in millimolar 124 NaCl, 3 KCl, 2.4 CaCl_2_, 2 MgCl_2_, 1.25 NaH_2_PO4, 26 NaHCO3, and 10 D-glucose and was equilibrated with 95 % O2 and 5 % CO_2_. Hippocampal slices (400-μm thick) were maintained in aCSF-filled holding chamber at room temperature for at least 1 h and individually transferred to the submerged glass bottom recording chamber, which was constantly perfused with aCSF (2 ml/min) at 30 °C. Field excitatory postsynaptic potential (fEPSP) was recorded with a metallic (Pt/Ir) electrode (FHC, Bowdoin, ME) from the stratum radiatum layer of Cornu ammonis 1 region of the hippocampus (CA1) area, and the Schaffer collateral afferents were stimulated with 100-μs test pulses via a bipolar cluster electrode (FHC). For long-term potentiation (LTP) experiments, the stimulus strength was set to elicit 40–50 % of the maximum fEPSP amplitude and test pulses were delivered to Schaffer collaterals once every 30 s. LTP was induced by 3-theta-burst stimulation (3-TBS) protocol (each burst consisted of four pulses at 100 Hz with a 200-ms interburst interval). Before 3-TBS or drug application, the responses were monitored for at least 10 min to ensure a stable baseline of fEPSP. To determine whether the magnitude of LTP differed significantly between groups, average responses during the last 20-min block of recordings (40–60 min after TBS) were compared. All drugs and chemicals were applied directly to the slice via bath perfusion, which allowed for a complete exchange of the perfusate in less than a minute and a half.

**Behavioral testing**

Morris Water Maze (MWM)

The MWM apparatus consisted of 2-m circular blue plastic pool filled with water (24–25°C), which was rendered opaque by the addition of non-toxic white paint. An escape platform (20 cm in diameter) was submerged 0.5 cm under the water level. Dark posters, different in shape (one per wall) provided distant landmarks. The behavior of a mouse was recorded by a video camera connected to a video tracking system (HVS Image 2100, HVS Image, Buckingham, UK).

The pool was surrounded by a white curtain, and a mouse was released facing the wall at points (N, E, S, W) which were chosen semi-randomly. The mice were trained for 3 days (4 trials per day) to find a submerged platform located in the center of the NE quadrant of the pool (target quadrant, TQ). The trial ended when a mouse found and climbed onto the platform within 120 s. If the mouse failed to find the platform, it was guided to the platform by an experimenter. After a 10-s post-trial time on the platform, the mouse was placed in a holding cage to dry. Mice were tested with inter-trial interval of 50 min. Memory was evaluated in probe trial, administered on day 4 as the first trial of the day. During probe trial, the platform was removed from the pool. Memory for the platform location was expressed as the percent of time spent in TQ.

**Immunofluorescent-Histological Staining**

After completion of treatments, all mice were sacrificed with an overdose of isoflourane anesthetic, perfused transcardially with saline using a pump, and the brains were harvested. The right hemisphere was frozen for biochemical analysis (Western blot, ELISA), and the left hemisphere was fixed with PAF for 24 h at 4 C. These brain tissues were further processed with modified CLARITY protocol (http://www.chunglabresources.com/clarity/). Briefly, the fixed brain tissue was transferred to hydrogel monomer solution (4% acrylamide in PBS) at 4 C for 24 h, and subsequently to a 24-well plate, merged in fresh hydrogel solution and the tissue brought to 37 C till formation of the gel. Thick sagittal slices (400 μm) were cut on an HR2 Slicer (Sigman Electronic, Germany). The thicker brain sections were cleared with 8% SDS in PBS for 24 h, followed by 0.3% Triton X-100 in PBS for 24 h. Blocking the section in 2%BSA-10% goat serum for 4h. A modified thioflavin-S staining was used for detecting Aβ plaques. Briefly, the brain sections were rinsed with distilled water, dropped with thioflavin S (0.0125% in 50% ethanol) for 5 min, further washing with 50% ethanol and water. The stained clear slices were mounted on a glass slide using Dow Corning high vacuum greese surround to cylinder shapes of a thickness slightly more than the thickness of slice. Images were visualized using fluorescence microscopy (Axioplan-2, Carl Zeiss Ltd). Amyloid plaque size and area were analyzed with Image J software.

**Western blot**

Frozen brain tissues or cultured cells were homogenized in cold RIPA buffer with protease inhibitors and proteins were quantified with BCA assay (BioRad, Mississauga, ON, Canada). Proteins were loaded at 50 μg per lane on a 12% polyacrylamide gel. Proteins were transferred to nitrocellulose membrane and then blocked with LiCor blocking buffer. Blots were further incubated with primary antibodies overnight at 4 °C on a shaker. Primary antibody used for 6E10 for APP (1:5000 mouse; 6E10; Covance), CD68 (1:500, mouse monoclonal, Dako), caspase-1 (1:1000, rabbit, Abcam), NLRP3 (1:1000, rabbit, Millipore), and β-actin (1:10,000 mouse, Sigma-Aldrich). IRDye 800CW goat anti-rabbit and IRDye 680CW goat anti-mouse (LiCor, 1:10,000) were used as secondary antibodies. Blots were imaged using LiCor Odyssey image system.

**Statistical analysis**

Statistical Analysis—Values are means ± S.D. Significance was determined using either student t test, or one-way analysis of variance, followed by Tukey’s test when appropriate, with Prism software (GraphPad Prism 5, GraphPad Software, San Diego, CA). p < 0.05 was considered significant.

**Supplementary Table S1**

| **Peptide** | **Sequence** | **MALDI-TOF MW [M+H]^+^** | | **Yield (%)** | **Purity (%)** |
| --- | --- | --- | --- | --- | --- |
|  |  | Calculation | Observation |  |  |
| R5 | SQELHRLQTYPR | 1527.5 | 1526.5 | 89 | 96 |
| R14 | LGRLSQELHRLQTY | 1713.1 | 1712.1 | 80 | 98 |
| R11 | LQTYPRTNTGSN | 1351.6 | 1351.8 | 75 | 95 |

**Table S1**: MALDI-TOF mass spectrum of peptide of the three selected peptides used in the study R5, R14, and R11.

**
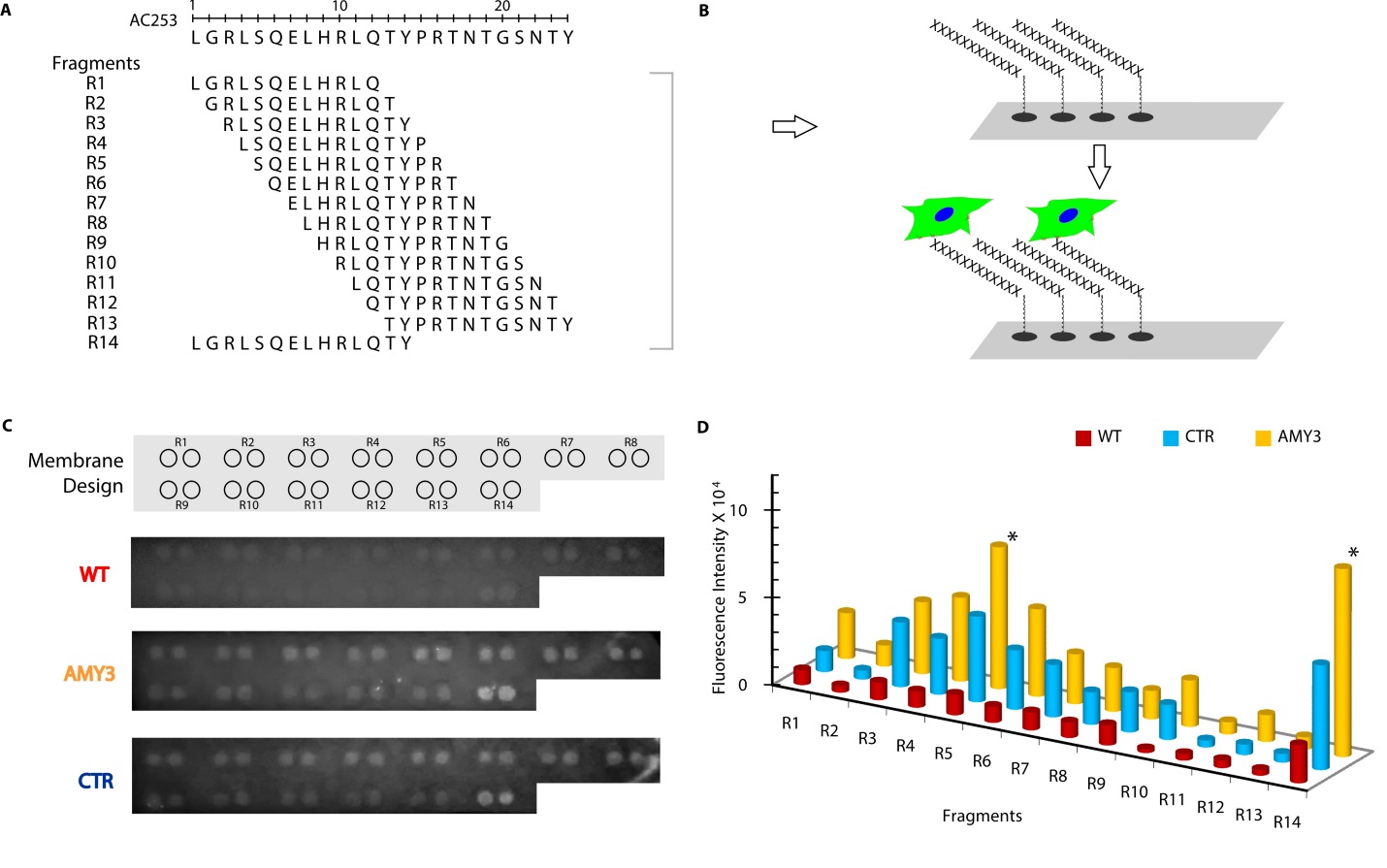
**

**Supplementary Figure S1**: **Design and synthesis of AC253 based peptide library.** **(A)** A peptide library of 14 peptide fragments (12-14 amino acids) was generated based on AC253 sequence. **(B)** The peptide library (12-14 amino acid) was synthesized onto cellulose membrane, each in duplicate, followed by incubation with GFP labeled cells for 4 h. **(C)** Screening of peptide cellulose membrane libraries with different GFP labeled cell lines are presented from AMY3 receptor expressing cells (yellow), HEK293 wild type cells (red), and calcitonin receptor (CTR) expressing cells (blue). Fluorescence intensity of peptide binding to cells was measured using a Kodak imager, with excitation and emission at 467 and 535 nm, respectively. **(D)** Quantification of fluorescence showed that peptides R5 and R14 have the most significant binding to AMY3 cells compared to other fragments. R11 has the least binding and was selected as negative control sequence for subsequent experiments.


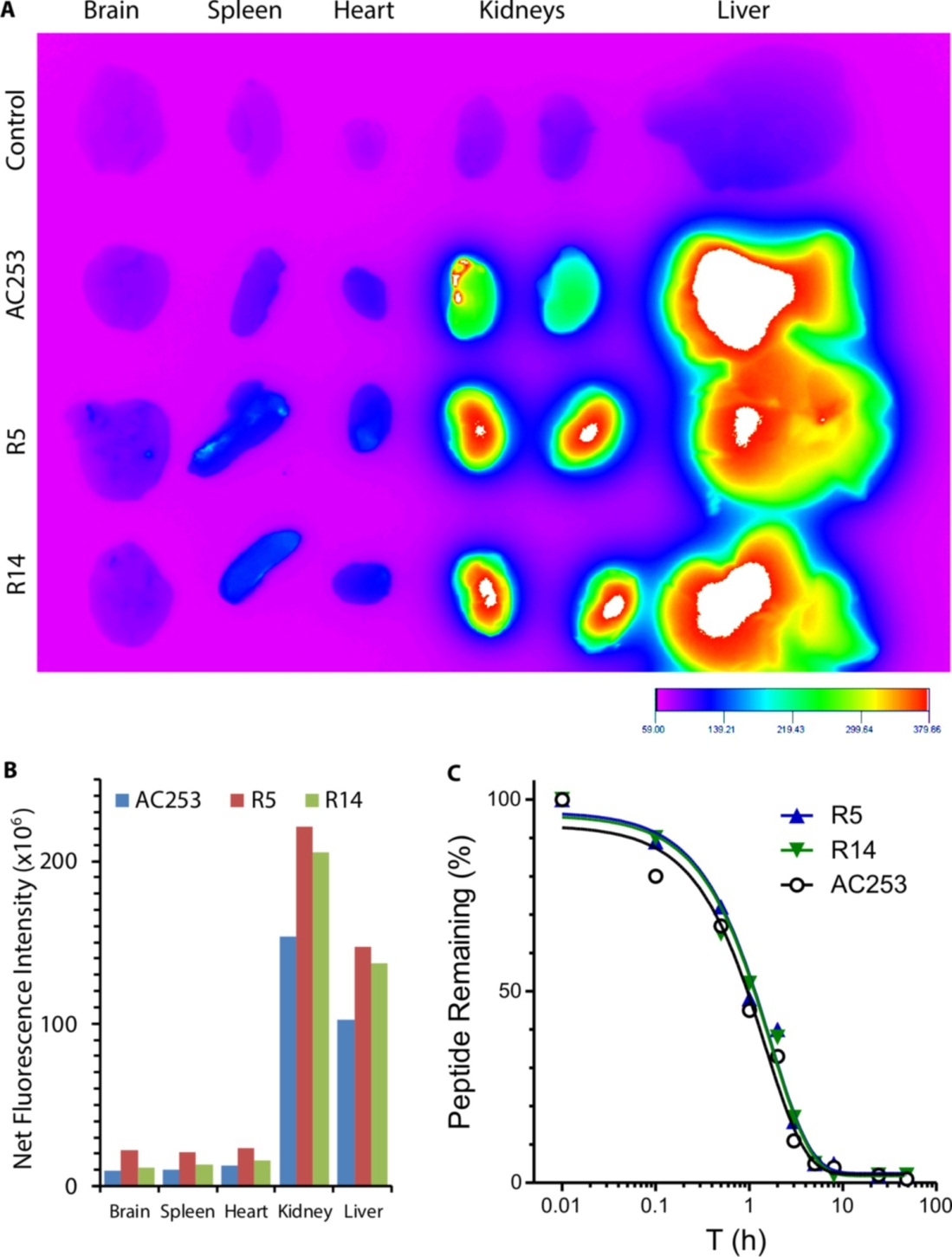


**Supplementary Figure S2**: **The comparison of bio-distribution for the fragments and their parent peptide.** (**A**) *Ex vivo* fluorescence showing bio-distribution images for Cy5.5-R5, R14, AC253 in different organs at after a 2 h of a single ip injection of the peptides (0.1 mmol in 200µl saline). (**B**) Quantification of peptides fluorescence in each organ (n=3). (**C**) In vitro stability of R5, R14, and AC253 peptides in human serum compared with AC253 at 37°C. The amount of intact peptide in human serum at different time points was estimated using RP-HPLC.


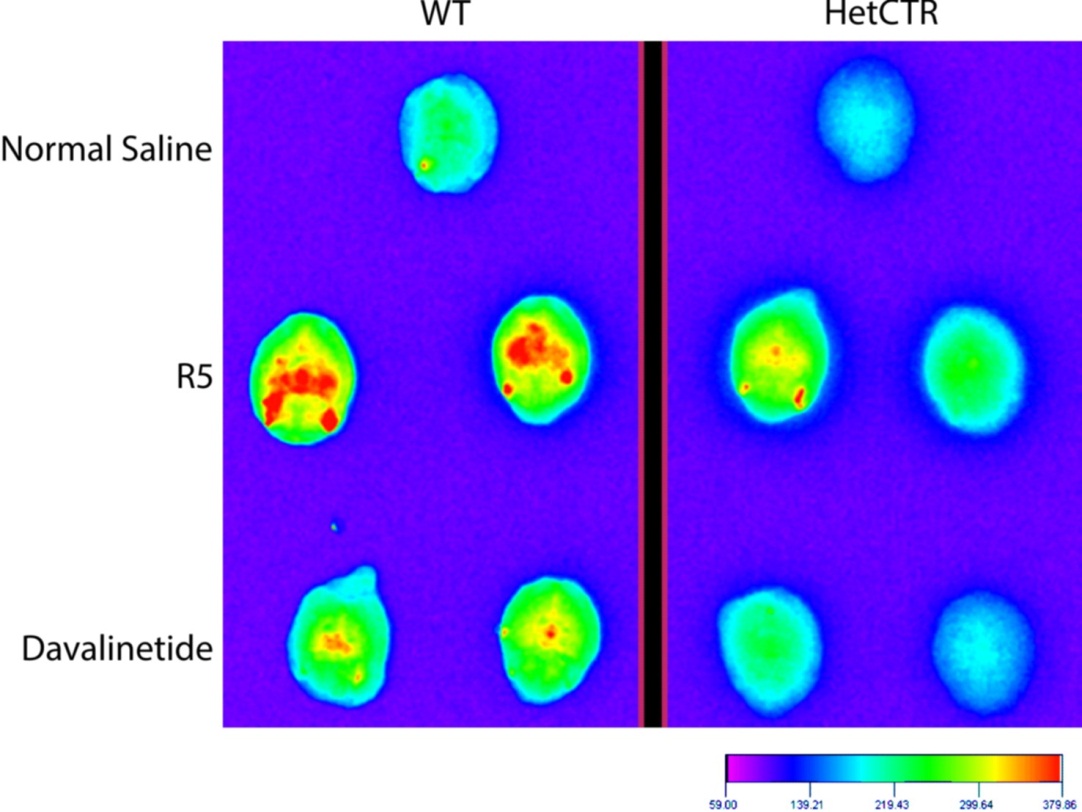


**Supplementary Figure S3: The comparison of brain permeability for R5 and Davalintide.** Imaging of the intact brain at 2 h post-injection of Cy5.5 labeled R5 or Davalintide (0.1mmole) showing superior brain permeability of R5 in comparison to Davalintide in wild-type (WT) and 50% amylin receptor depleted heterozygous CTR (HetCTR) mice.


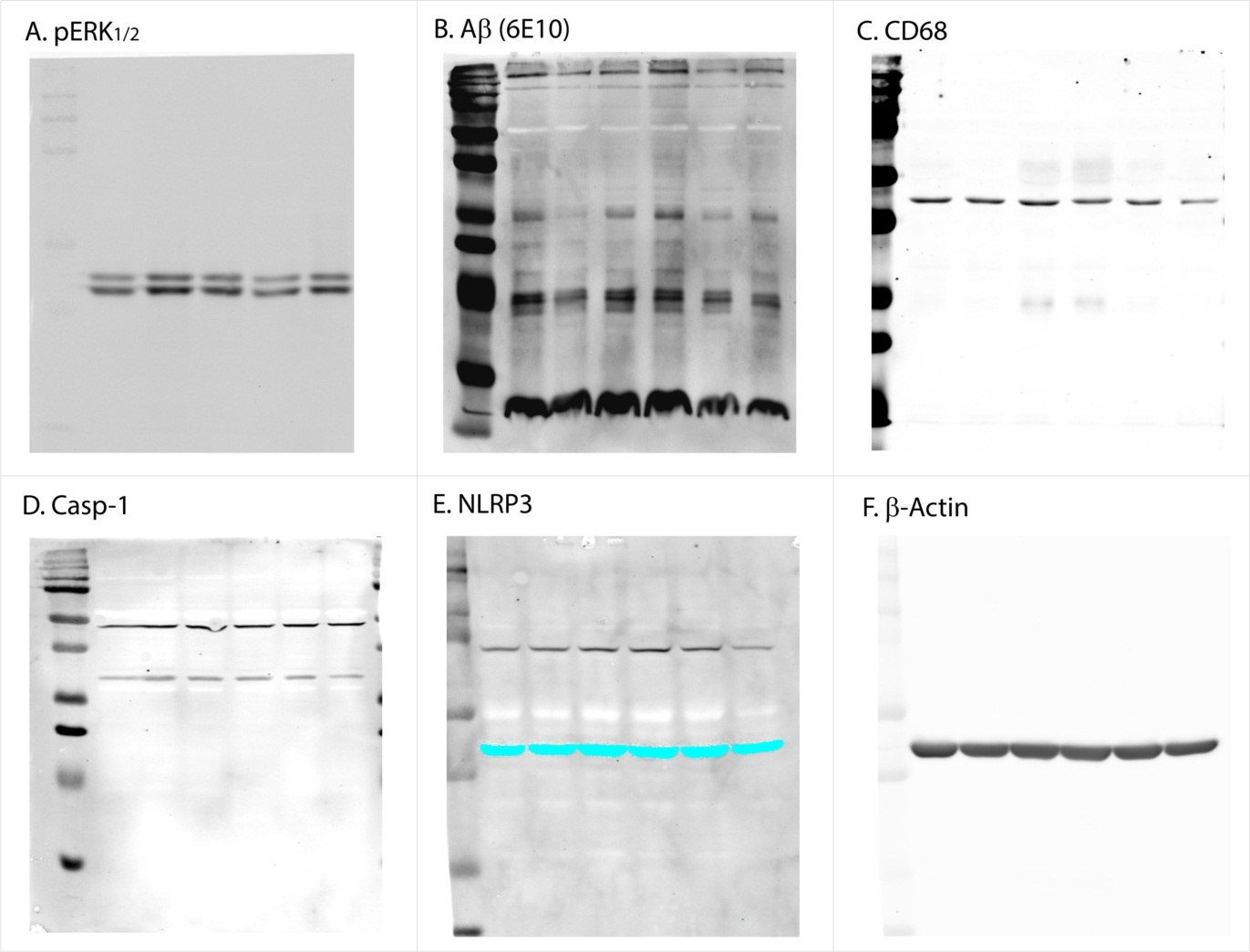


**Supplementary Figure S4:** The list of full membranes for Western Blot in Figure 1F (**A**) and Figure 5C (**B-F**).
